# High PEEP in acute respiratory distress syndrome: quantitative evaluation between improved arterial oxygenation and decreased oxygen delivery

**DOI:** 10.1093/bja/aew314

**Published:** 2016-10-31

**Authors:** M. Chikhani, A. Das, M. Haque, W. Wang, D. G. Bates, J. G. Hardman

**Affiliations:** 1 Anaesthesia and Critical Care Section, Division of Clinical Neuroscience, School of Medicine, University of Nottingham, Nottingham NG7 2UH, UK; 2 Nottingham University Hospitals NHS Trust, Nottingham NG7 2UH, UK; 3 School of Engineering, University of Warwick, Coventry CV4 7AL, UK

**Keywords:** computer simulation, respiration, artificial, respiratory distress syndrome, adult

## Abstract

**Background.** Positive end-expiratory pressure (PEEP) is widely used to improve oxygenation and prevent alveolar collapse in mechanically ventilated patients with the acute respiratory distress syndrome (ARDS). Although PEEP improves arterial oxygenation predictably, high-PEEP strategies have demonstrated equivocal improvements in ARDS-related mortality. The effect of PEEP on tissue oxygen delivery is poorly understood and is difficult to quantify or investigate in the clinical environment.

**Methods.** We investigated the effects of PEEP on tissue oxygen delivery in ARDS using a new, high-fidelity, computational model with highly integrated respiratory and cardiovascular systems. The model was configured to replicate published clinical trial data on the responses of 12 individual ARDS patients to changes in PEEP. These virtual patients were subjected to increasing PEEP levels during a lung-protective ventilation strategy (0–20 cm H_2_O). Measured variables included arterial oxygenation, cardiac output, peripheral oxygen delivery, and alveolar strain.

**Results.** As PEEP increased, tissue oxygen delivery decreased in all subjects (mean reduction of 25% at 20 cm H_2_O PEEP), despite an increase in arterial oxygen tension (mean increase 6.7 kPa at 20 cm H_2_O PEEP). Changes in arterial oxygenation and tissue oxygen delivery differed between subjects but showed a consistent pattern. Static and dynamic alveolar strain decreased in all patients as PEEP increased.

**Conclusions.** Incremental PEEP in ARDS appears to protect alveoli and improve arterial oxygenation, but also appears to impair tissue oxygen delivery significantly because of reduced cardiac output. We propose that this trade-off may explain the poor improvements in mortality associated with high-PEEP ventilation strategies.

Editor’s key points

- Positive end-expiratory pressure is widely used in mechanically ventilated patients with the acute respiratory distress syndrome (ARDS), but the effect of PEEP on tissue oxygen delivery is not known.
- The authors investigated the effects of PEEP on tissue oxygen delivery in ARDS.
- Increasing PEEP increased arterial oxygen tension but decreased tissue oxygen delivery.

The incidence of acute respiratory distress syndrome (ARDS) has been estimated at ∼70 per 100 000 patients yr^−1^,^1^^,^^2^ with a lethal outcome in 55% of patients.^3^ The global burden of the disease was estimated as 5.5 million patients yr^−1^ requiring intensive care unit admission and mechanical pulmonary ventilation.^4^

The rationale for the application of PEEP during mechanical ventilation of the lungs of patients with ARDS is to prevent alveolar collapse, reducing injurious alveolar shear stresses and improving ventilation–perfusion matching, and thus, arterial oxygenation.^5^^,^^6^ Studies investigating the effect of PEEP have consistently shown an improvement in oxygenation and pulmonary compliance.^5^^,^^7–10^ Survival benefit was seen in patients when PEEP was assigned based on oxygen requirements in combination with low *vs* traditional tidal volume ventilation,^11^ and some risk reduction was shown in a pooled subgroup analysis when patients were stratified based on ARDS severity.^12^ Despite this, the results of four large studies examining high-PEEP strategies in ARDS have demonstrated an equivocal effect on mortality,^13–16^ confirmed after meta-analysis.^12^ Consequently, it is unclear who might benefit from the application of PEEP; clinical investigation has, to date, failed to provide a conclusive answer.

High levels of positive airway pressure throughout the respiratory cycle have the potential to impair cardiac performance, manifested as a reduced cardiac output.^17–20^ This is a result of increased right ventricular afterload, reduced left ventricular preload, and reduced biventricular compliance.^21^ It is credible that PEEP-induced reduction in cardiac output may outweigh the benefit of improved arterial oxygenation, resulting in reduced organ oxygen delivery ($D_{O_{2}}$), and this was suggested by the early work by Suter and colleagues^22^ examining the effect of PEEP on lung compliance. Given the difficulty of accurately measuring oxygen delivery *in vivo*, arterial oxygenation is the usual, clinical target for ventilatory optimization; thus, we may remain unaware of the quantitative effect that PEEP might have with respect to oxygen delivery.

Acute respiratory distress syndrome is a heterogeneous disease process, with widely varying cause and progression. This makes it an ideal candidate for high-fidelity modelling studies that can investigate the benefits and hazards of PEEP across individuals in the safe, cost-effective, reproducible *in silico* environment.

## Methods

Our study uses a highly integrated computer simulation model of the pulmonary and cardiovascular systems that has recently been developed by our group.^23^^,^^24^ The model includes 100 independently configured alveolar compartments and 19 cardiac compartments. Aspects of the model related to pulmonary pathophysiology have been validated previously,^23^^,^^25–28^ including ARDS.^29^^,^^30^ This model was integrated with a multicompartmental, contractile cardiovascular model with pulsatile blood flow and ventilation-affected, transalveolar blood flow. The cardiac section of the model consists of two contractile ventricles, with atria modelled as non-contractile, low-resistance, high-compliance compartments. The mathematical principles underpinning the model are detailed in the Supplementary appendix.

Cardiopulmonary interactions are modelled in a number of ways. Ventricular contractility is modelled as a truncated sine-wave varying ventricular elastance over time. Intrapulmonary pressure is transmitted variably across ventricular walls such that lung inflation reduces ventricular compliance; in this study, 85% of intrapulmonary pressure was transmitted across the ventricles. Transalveolar blood flow is governed by pulmonary arterial pressure and by transalveolar vasoresistance; this resistance is affected dynamically in each alveolar compartment by alveolar volume (causing longitudinal stretch) and alveolar pressure (causing compression).

Published, clinical data were used to validate the responses of the integrated model against those of individual ARDS patients. Global optimization algorithms were used to configure the model parameters (e.g. microbronchial resistances, transalveolar vasoresistances, and alveolar compliance) against published clinical trial data on tidal volume, respiratory rate, haemoglobin concentration, metabolic rate, and pulmonary shunt fraction, in order to replicate arterial blood gas values. Once these static configurations were determined, cardiovascular settings in the model (e.g. ventricular contractility, compartmental blood volumes, arterial resistances, and ventricular splinting) were manually tuned to match reported dynamic changes in cardiovascular performance during PEEP variation. Where there were deficiencies in the published data sets, historically appropriate patient characteristics and clinical data were used to create a plausible estimate of the missing data values. For example, if haemoglobin concentration was not reported, it was estimated to be 100 g litre^−1^ (this being a common clinical target before the publication of the TRICC study in 1999);^31^ where ventilation mode was not specified, it was assumed to be volume controlled with a constant inspiratory flow and inspiratory-to-expiratory ratio of 1:2 (this being reported in many clinical trials investigating ventilation strategies for ARDS).^11^

Initial matching was against data derived from a single ARDS patient reported by Dantzker and colleagues.^17^ This patient had severe ARDS, with arterial oxygen partial pressure ($Pa_{O_{2}}$)-to-fractional inspired O_2_ ratio of 87 mm Hg (11.6 kPa), and underwent a four-stage incremental PEEP trial. Thereafter, the same process was carried out using the clinical data sets of Jardin and colleagues^18^ and Pinsky and colleagues;^20^ for each of these clinical studies, the model was matched to the average cardiopulmonary state of each study population and subjected to the published incremental PEEP trials. Model outputs were compared against the data collected in the clinical studies.

Model simulation and comparison of results with the historical data were carried out by two independent investigators in different universities, allowing corroborated evaluation of the simulations. Full details of the methodology used in validating the model against clinical data are provided in the Supplementary appendix.

When accurate reproduction of results was achieved, we were able to proceed to prospective testing of the effects of an incremental PEEP trial on $D_{O_{2}}$ in a variety of matched *in silico* subjects with various ARDS disease configurations.

### PEEP trial simulation protocol

An *in silico* bank of 12 ARDS subjects was created by configuring parameters in the model to match published pathophysiological data from several publications.^17^^,^^18^^,^^20^^,^^32^^,^^33^ An additional ‘healthy’ subject was configured to provide a baseline response (Table S3 in the Supplementary appendix).

The cardiovascular model parameters used in the validation process were used to initialize the cardiovascular system model for prospective testing, and these parameters are reported in the Supplementary appendix (Table S6).

Modelled patients received pulmonary ventilation using settings in line with recommendations from the ARDSnet study;^11^ using square-wave pressure-controlled ventilation, with a constant inspiratory-to-expiratory ratio of 1:2 and ventilatory rate of 10 bpm. Inspiratory pressure was adjusted to maintain a tidal volume between 450 and 600 ml (6–8 ml kg^−^^1^) to maintain arterial carbon dioxide partial pressure ($Pa_{{\text{CO}}_{2}}$) between 4 and 10 kPa based on the findings of early tidal volume and permissive hypercapnia studies.^34–36^ The initial fractional inspired O_2_ from the matching process was fixed and kept constant throughout; this ensured that any observed increase in oxygenation would be as a result of alveolar recruitment. The PEEP started at 0 cm H_2_O and increased by 5 cm H_2_O every 10 min to a maximum of 20 cm H_2_O, before finally returning to 0 cm H_2_O (see Table 1).

**Table 1:** Summary of findings and trial protocol. Data presented are the mean average (sd) values at each PEEP value for all 12 simulated patients. CO, cardiac output; $D_{O_{2}}$, oxygen delivery; MAP, mean aortic arterial pressure; $Pa_{{\text{CO}}_{2}}$, arterial partial pressure of carbon dioxide; $Pa_{O_{2}}$, arterial partial pressure of oxygen; $Sa_{O_{2}}$, arterial oxygen saturation; $Sv_{O_{2}}$, mixed venous oxygen saturation; VT, tidal volume

Parameter	PEEP 0 cm H_2_O	PEEP 5 cm H_2_O	PEEP 10 cm H_2_O	PEEP 15 cm H_2_O	PEEP 20 cm H_2_O	PEEP 0 cm H_2_O
VT (ml)	488 (68.6)	503 (63.3)	507 (78.9)	520 (74.4)	545 (72.6)	488 (68)
MAP (mm Hg)	89.4 (2.12)	85.4 (1.70)	82.2 (1.22)	79.4 (1.05)	76.9 (0.99)	89.3 (2.11)
CO (ml min^−1^)	6523 (197)	6008 (153)	5552 (95.8)	5106 (77.4)	4669 (74.3)	6520 (199)
$Sa_{O_{2}}$ (%)	89.2 (7.26)	90.2 (6.26)	89.5 (7.17)	93.1 (7.52)	93.1 (8.47)	89.1 (7.33)
$Pa_{O_{2}}$ (kPa)	8.90 (2.88)	9.54 (2.99)	9.86 (3.79)	11.25 (3.86)	15.8 (8.78)	9.36 (2.77)
$Pa_{{\text{CO}}_{2}}$ (kPa)	5.63 (0.99)	6.26 (1.54)	6.61 (1.83)	6.59 (1.89)	6.37 (1.96)	6.80 (1.92)
$Sv_{O_{2}}$ (%)	56.0 (1.54)	54.3 (1.50)	50.6 (1.66)	50.0 (1.75)	48.3 (2.00)	56.0 (1.54)
$D_{O_{2}}$ (ml min^−1^)	792 (271)	736 (242)	676 (223)	636 (200)	591 (185)	791 (272)
Recruitment (%)	66.2 (12.8)	70.3 (12.3)	73.0 (12.4)	78.1 (11.2)	83.7 (11.4)	66.3 (12.8)
Dynamic strain (ratio)	0.74 (0.28)	0.60 (0.18)	0.56 (0.17)	0.49 (0.16)	0.43 (0.18)	0.77 (0.27)

The following outputs were recorded every 10 ms: arterial haemoglobin oxygen saturation ($Sa_{O_{2}}$), mixed venous haemoglobin oxygen saturation ($Sv_{O_{2}}$), $Pa_{O_{2}}$, $Pa_{{\text{CO}}_{2}}$, arterial pH, alveolar recruitment (the fraction of alveoli receiving non-zero ventilation), cardiac output, aortic blood pressure, arterial oxygen delivery ($D_{O_{2}}$), and dynamic alveolar strain (as a surrogate for the risk of alveolar injury) as suggested by Protti and colleagues.^37^ Data are presented as averages throughout the ninth minute after each change in PEEP (i.e. during the minute preceding the next PEEP value).

Model simulations were run on a 64-bit Intel Core i7 3.7 GHz Windows 7 personal computer, running Matlab version R2014a (8.3.0.532; MathWorks Inc., Natick, MA, USA). Research ethics committee approval was not sought, because the data used for model development and validation were sourced from studies that had already received ethical approval. Simulation protocols were performed purely *in silico* without participation from human subjects.

## Results

Results of the initial calibration are shown in the Supplementary appendix; Fig. S4 shows the close fit of the model to data from patient-8 from Dantzker and colleagues^17^ against cardiac output, ventricular stroke volume, pulmonary vascular resistance, and $Pa_{O_{2}}$. Figures S5 and S6 in the Supplementary appendix show simulation performance against the clinical results from Jardin and colleagues^18^ and Pinsky and colleagues,^20^ respectively. Modelled results were consistently very close to those of clinical studies, indicating acceptable validity of the models in reproducing dynamic, *in vivo*, multi-organ behaviour.

Table 1 shows the average value of each measured parameter in all 12 *in silico* subjects at each PEEP setting during the implemented PEEP trial simulation protocol. The results presented show the mean and sd for all 12 *in silico* subjects at each PEEP setting for each reported parameter. Figures displaying the behaviour of each subject in the group are provided in the Supplementary appendix.

**Fig 1:**
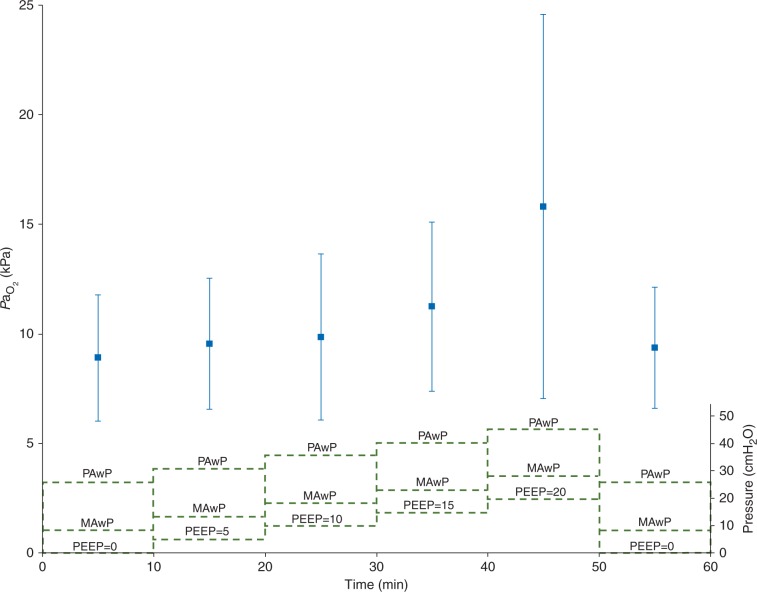
Arterial partial pressure of oxygen ($Pa_{O_{2}}$) over time during incremental PEEP trial. Filled blocks with errors bars represent group mean and sd for all 12 *in silico* patients (left axis). Dashed lines show the average ventilation metrics during the incremental PEEP trial (PaWP, peak airway pressure; MaWP, mean airway pressure; right axis).

**Fig 2:**
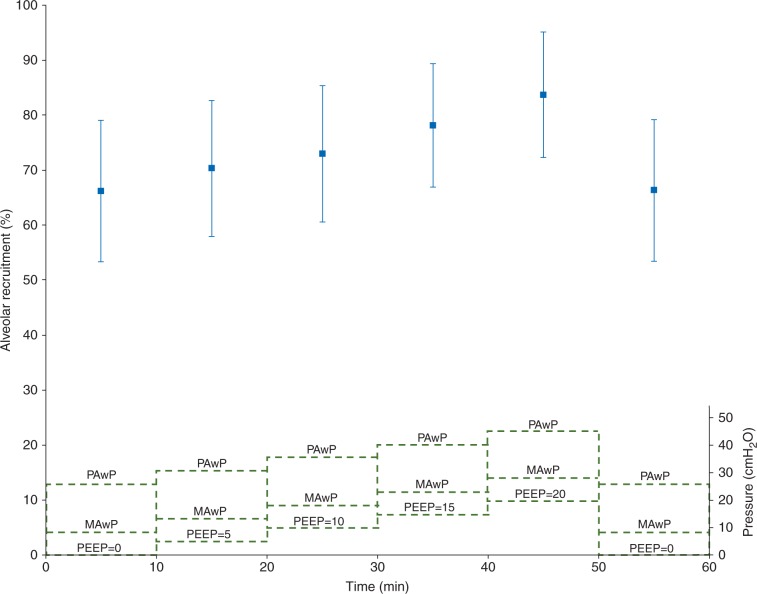
Percentage of recruited alveolar compartments over time during incremental PEEP trial. Filled blocks with errors bars represent group mean and sd for all 12 *in silico* patients.

**Fig 3:**
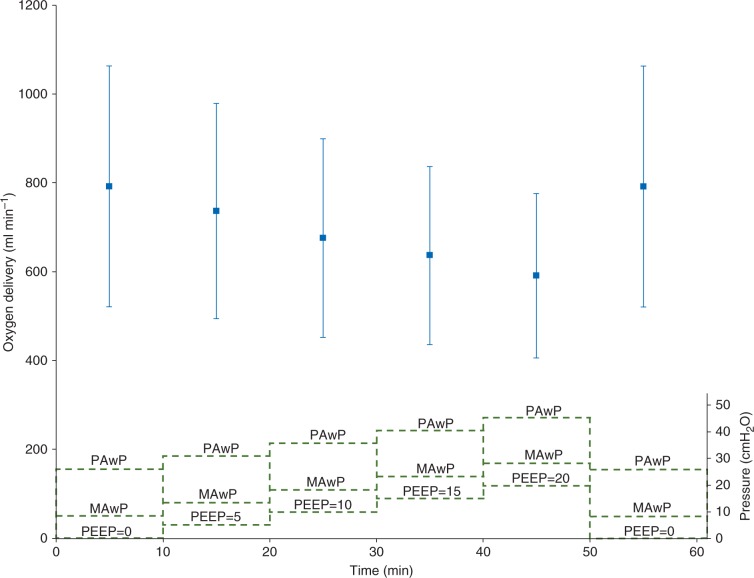
Oxygen delivery over time during incremental PEEP trial. Filled blocks with errors bars represent group mean and sd for all 12 *in silico* patients.

In all but the healthy subject, PEEP increased arterial oxygenation. At 20 cm H_2_O PEEP, in comparison with the value at 0 cm H_2_O PEEP, the following changes were observed; each is expressed as mean (sd, range): $Pa_{O_{2}}$ increased by 6.9 kPa (8.77, −0.47 to 27.0 kPa; Fig. 1); $Sa_{O_{2}}$ increased by 3.9% (6.36, −4.33 to 19.4 kPa); recruited alveolar compartments increased by 18% (10.3, 0–42.8%; Fig. 2); mean arterial pressure reduced by 22 mm Hg (1.79, 9.20–15.5 mm Hg); cardiac output reduced by 1.85 litres min^−1^ (0.17, 1.46–2.11 litres min^−1^); and oxygen delivery reduced by 0.20 litres min^−1^ (0.20, 0.07–0.46 litres min^−1^; Fig. 3).

**Fig 4:**
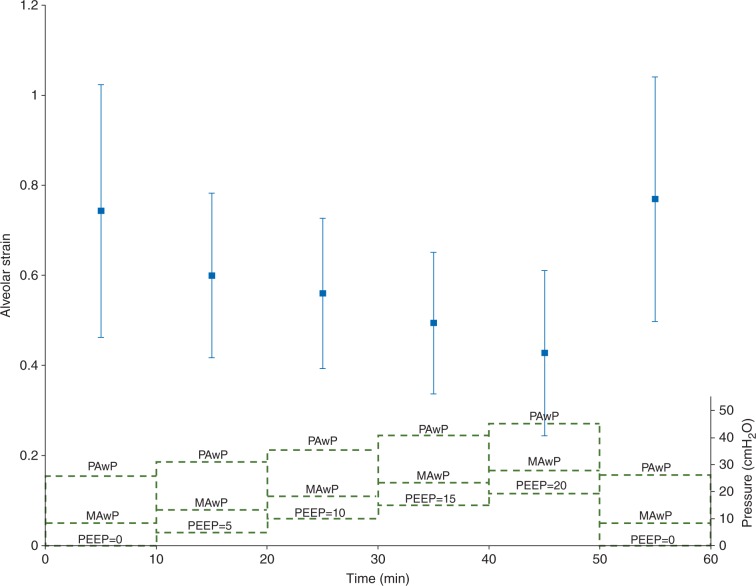
Dynamic lung strain over time during incremental PEEP trial. Filled blocks with errors bars represent group mean and sd for all 12 *in silico* patients.

Average dynamic alveolar strain (i.e. alveolar tidal volume/end-expiratory volume) decreased with the incremental addition of PEEP, with an absolute average reduction in strain ratio of 0.314, representing a relative average reduction in strain of 43% across the group; however, the exact relationship between PEEP and strain varied within the group (Fig. 4). The relationship between alveolar unit recruitment and dynamic alveolar strain ratio for all PEEP settings in all 12 *in silico* subjects is shown in Fig. S7 in the Supplementary appendix.

## Discussion

The use of a highly integrated pulmonary and cardiovascular computer simulation allowed systematic investigation of the effects of incremental PEEP on the cardiopulmonary systems in an *in silico* population of ARDS patients.

*In silico* simulation allows repeated, systematic investigation, without confounding by unquantified (‘silent’) interpatient variability or variability over time. The use of a population of modelled subjects allows greater confidence in extrapolating our findings to patients. The standardization allowed by an *in silico* population reduced ‘data noise’, and we anticipate that pragmatic and ethical issues would prevent a similar investigation *in vivo*.

From previous work,^30^ it was expected that improved oxygenation would arise from alveolar recruitment. There is currently no standardized duration for a clinical trial of incremental PEEP; however, there are recommendations for the duration of recruitment manoeuvres lasting from a few seconds^38^ to several minutes.^5^^,^^6^ It was observed that after ventilation changes, the model required a period of equilibration, during which alveolar compartment recruitment occurred, and cardiac performance and gas exchange stabilized. On this basis, the duration of each level of PEEP was set at 10 min, approximately twice the time required for 98% equilibration.^24^

Increasing PEEP markedly increased $Pa_{O_{2}}$; however, the accompanying increase in haemoglobin saturation was smaller, reflecting the finite oxygen-binding capacity of haemoglobin. The greatest increase in $Pa_{O_{2}}$ was observed in patients with the worst hypoxaemia at 0 cm H_2_O PEEP, in whom the supplemental airway pressure caused the greatest recruitment of collapsed alveoli (Fig. 2); this was supported by the demonstration that PEEP-induced improvement in oxygenation reversed when PEEP returned to 0 cm H_2_O.

Cardiac output decreased relatively consistently in all subjects in response to incremental PEEP. This was attributable to a combination of ventricular splinting by the distended lung, reducing right ventricular preload, and increased pulmonary vascular resistance, increasing right ventricular afterload; both serving to reduce right ventricular ejection and left ventricular filling.^24^ In all patients, the PEEP-induced reduction in cardiac output outweighed the induced improvement in oxygenation, such that increasing PEEP decreased $D_{O_{2}}$ in every modelled circumstance.

There was a decrease in dynamic strain ratios during the PEEP trial, with return to baseline levels on removal of PEEP. The degree of dynamic strain reduction appeared to be related to the presence of collapsed but recruitable alveolar units, and this is best illustrated graphically, where strain is plotted against recruitment (Fig. S7 in the Supplementary appendix). Examination of the individual patient data shows that an increase in PEEP caused strain to increase, until the threshold opening pressure was achieved for the collapsed lung units; when new alveolar units opened, the total distending force was distributed amongst more alveoli, thereby reducing the average strain for the whole lung.

Death in patients with ARDS rarely appears to be attributable to respiratory failure *per se*; rather, the majority of deaths are attributable to the underlying ARDS trigger or disease progression to systemic inflammatory response syndrome or multiple organ failure.^39^ This is caused in part by biological lung trauma (‘biotrauma’) caused by alveolar epithelial damage, resulting in the release of pulmonary cytokines,^39^ but may also be compounded by systemic release of inflammatory cytokines secondary to inadequate organ perfusion.

Findings from this investigation offer an explanation for the apparent lack of mortality benefit in studies examining high-PEEP strategies in patients with ARDS,^13–16^ and for the fact that subgroup analyses indicate that those with the severest disease may benefit most from PEEP.^12^ Careful examination of each *in silico* patient demonstrates that those with the worst starting hypoxaemia and greatest number of recruitable alveolar compartments exhibited the greatest improvement in oxygenation compared with reduction in cardiac output. Likewise, those with the largest number of recruitable compartments also exhibited the greatest reduction in the average dynamic lung strain.

The recent mediation analysis of large randomized control studies by Amato and colleagues^40^ has suggested that high-PEEP strategies may not always be protective, and high plateau pressures may not necessarily add mortality risk in ARDS. The protocol for our modelling study was based on stepwise escalation of PEEP with fixed driving pressure (using a pressure-controlled mode of ventilation), and we were therefore unable to investigate variation in our recordings based on fixed PEEP *vs* matching plateau pressure. However, the results do demonstrate different proportions of alveolar compartment recruitment and dynamic lung strain on an individual patient basis for an almost uniform set of driving pressures across the group. This is most evident in Figs S25, S26, and S27 in the Supplementary appendix. Modelling may provide the ideal opportunity to examine further the variation in driving pressure during ventilation in ARDS and its relationship to oxygenation, ventilation, oxygen delivery, and lung mechanics.

Our demonstration of a consistent decrease in $D_{O_{2}}$ in response to PEEP in an *in silico* population is novel. The influence of PEEP on cardiovascular performance outweighed, in every patient, the improvement in oxygenation in an experimental population with deliberately varied ventilation–perfusion mismatching and alveolar collapse. The notion that PEEP can be categorized in terms of improvement in oxygenation, reduction of shunt fraction, and impact on the cardiovascular system is not in itself new.^41^ There seems to be agreement that PEEP should be an addition to ventilation strategy in ARDS,^42–44^ although there is still much debate concerning the determination of the correct level of PEEP for use in patients with ARDS.^45^ Titration of PEEP to cardiovascular performance requires estimation of the cardiac output; however, the use of the ‘gold-standard’ pulmonary artery catheter has decreased dramatically over the last decade,^46^^,^^47^ and despite surveys of practice indicating the facility to use flow monitoring in critical care, only a small proportion of critical care units seem to do so on a routine basis.^48^

Our study had several limitations that should be noted. The model was calibrated and validated against data from historical studies. It is plausible that the cardiovascular side-effects of historical drugs and dosage required to produce levels of sedation deep enough to allow the traditional high tidal volume ventilation, with or without neuromuscular block, may completely obtund normal cardiovascular system baroreceptor reflexes. Indeed, the aforementioned historical studies consistently report that heart rate did not change significantly throughout the duration of their interventions;^17–20^ it was therefore deemed appropriate to fix the heart rate at 100 beats min^−1^ for this investigation.

The importance of atrial contraction for ventricular filling is poorly understood. The majority of experimental data relate to lower mammalian studies, human studies with small numbers of volunteers, and stable patients, with possible confounding factors from study design and timing of follow-up.^49^ Consequently, in this study we used a lumped model of atrial and ventricular contraction; in the context of a fixed heart rate and the assumption of sinus rhythm and normal heart valves, we feel that this has minimal impact on the applicability of our results.

## Conclusion

The highly integrated cardiopulmonary model used in this study was able to match accurately the cardiorespiratory interactions of individual patients with ARDS receiving mechanical ventilation, allowing in-depth and controlled investigation of key outcome parameters using data that may not be routinely scrutinized in daily clinical practice.

Our results show that changing the ventilation strategy to improve commonly monitored oxygenation indices and increase alveolar protection by preserving the open lung may, counterintuitively, be at the hidden expense of reducing global oxygen delivery, particularly in patients with less severe ARDS. In clinical practice, PEEP-response trials should ideally include measurement and titration to $D_{O_{2}}$ in order to allow personalized application of optimal PEEP to maximize alveolar protection while minimizing the reduction in global oxygen delivery. Such a personalized approach might yield substantial improvements in the effectiveness of our existing therapeutic strategies.

## Authors’ contributions

Design of pulmonary model: J.G.H. 

Pulmonary model development and modelling of disease: A.D., W.W., D.G.B., J.G.H. 

Development and integration of cardiovascular model: M.H., J.G.H., A.D., M.C. 

Patient matching: A.D., W.W., D.G.B. 

Writing and final approval of manuscript: M.C., A.D., M.H., W.W., D.G.B., J.G.H.

## Declaration of interest

None declared. 

## Funding

UK Medical Research Council (grant number MR/K019783/1).

## Supplementary material

Supplementary material is available at *British Journal of Anaesthesia* online.
